# A novel fluoroscopic system improves visibility of devices during endoscopic ultrasound-guided pancreatic duct drainage

**DOI:** 10.1055/a-2414-7649

**Published:** 2024-09-26

**Authors:** Takeshi Ogura, Yuki Uba, Nobuhiro Hattori, Kimi Bessho, Hiroki Nishikawa

**Affiliations:** 1 38588 Endoscopy Center, Osaka Medical and Pharmaceutical University Hospital, Osaka, Japan; 2 13010 2nd Department of Internal Medicine, Osaka Medical and Pharmaceutical University, Osaka, Japan

Endoscopic ultrasound-guided pancreatic duct drainage (EUS-PDD) can be indicated for patients with pancreatic duct obstruction 1 2 3 4 . Compared with EUS-guided drainage for the biliary tract, such as gallbladder drainage or choledochoduodenostomy, improving the visibility of devices under fluoroscopic guidance is important because the main pancreatic duct is thin, and a plastic stent, which can have poor visibility on fluoroscopy, is usually used. To improve the fluoroscopic visibility of devices, a novel visibility enhancement mode of a fluoroscopic system (Astorex i9; Canon Medical Systems, Kanagawa, Japan), called “Accent mode,” has become available. Technical tips for EUS-PDD using Accent mode are presented.

A 59-year-old man was admitted to our hospital due to frequent obstructive pancreatitis. He underwent pancreatectomy for distal cholangiocarcinoma one year earlier. Because no findings of recurrence were observed around the pancreaticojejunostomy site on computed tomography (CT), a benign pancreaticojejunostomy stricture was suspected as the reason for pancreatitis. Therefore, EUS-PDD was attempted. First, the main pancreatic duct was identified, and puncture with a 19-G needle using color Doppler was performed to avoid vessel injury. Then, the contrast medium was injected. On pancreatography, a pancreaticojejunostomy stricture was observed ( Fig. 1 ). Insertion of a 0.025-inch guidewire (J-Wire NM; JMIT, Shiga, Osaka, Japan) was attempted, but the visibility of the guidewire was not very good ( Fig. 2 ) because the main pancreatic duct was not very dilated. Therefore, guidewire manipulation was performed in Accent mode. By doing so, the visibility of the guidewire was improved ( Fig. 3 ). After tract dilation was performed using a drill dilator (Tornus ES; Asahi Intecc, Aichi, Japan), the delivery system of a 7-Fr plastic stent (REGLUS; Japan Lifeline Co., Ltd., Tokyo, Japan) was inserted. However, the visibility of the plastic stent was also not very good ( Fig. 4 ). Therefore, Accent mode was used. After switching fluoroscopic modes, the visibility of the plastic stent improved ( Fig. 5 ), and deployment was successfully performed without any adverse events ( Video 1 ).

**Fig. 1:**
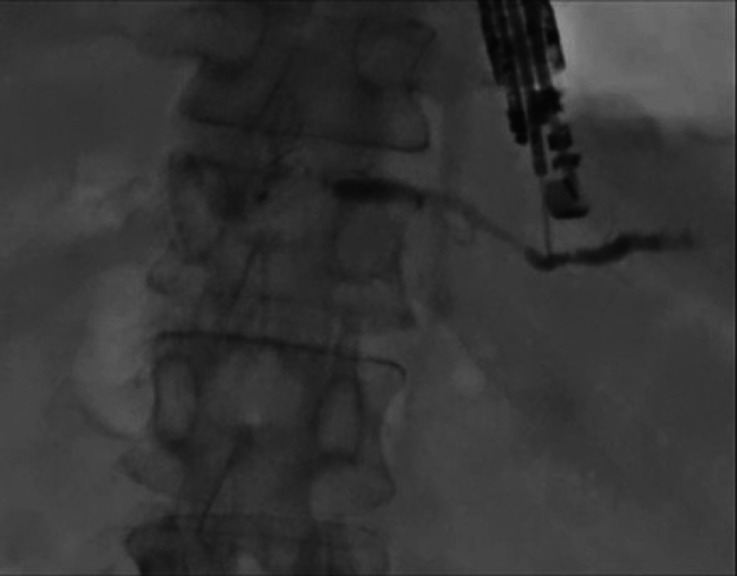
On pancreatography, a pancreaticojejunostomy stricture was observed.

**Fig. 2:**
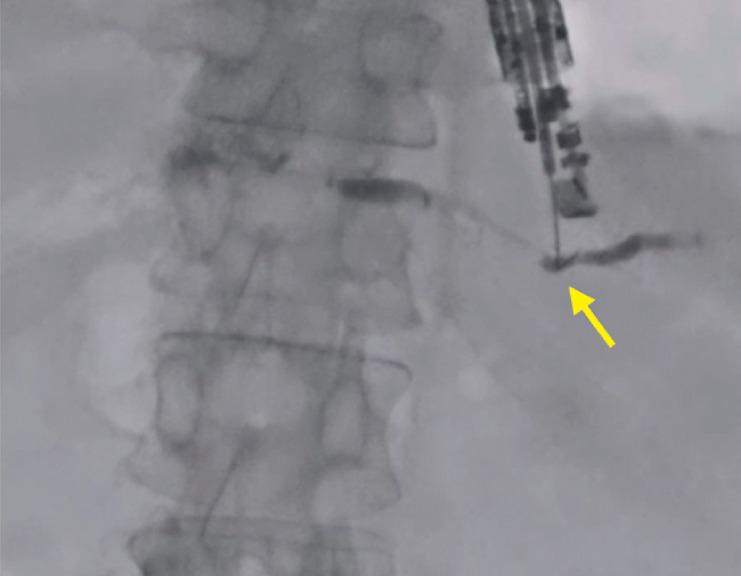
Insertion of a 0.025-inch guidewire was attempted, but the visibility of the guidewire was not very good (arrow).

**Fig. 3:**
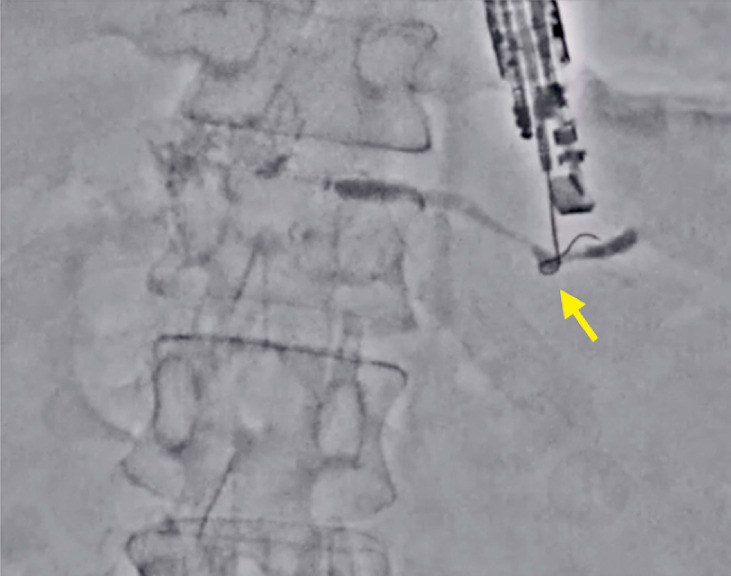
The visibility of the guidewire improved in Accent mode (arrow).

**Fig. 4:**
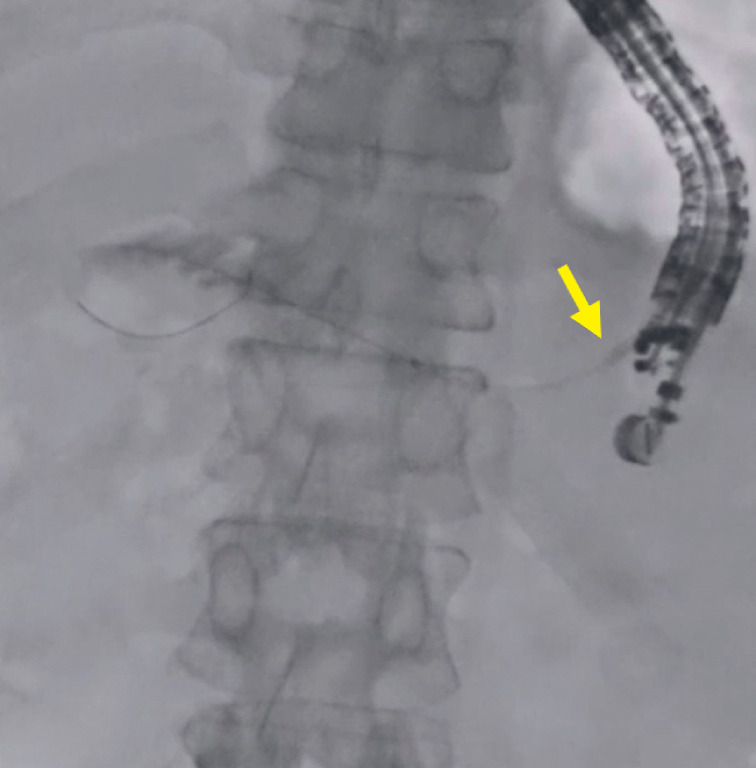
The visibility of the plastic stent was not very good (arrow).

**Fig. 5:**
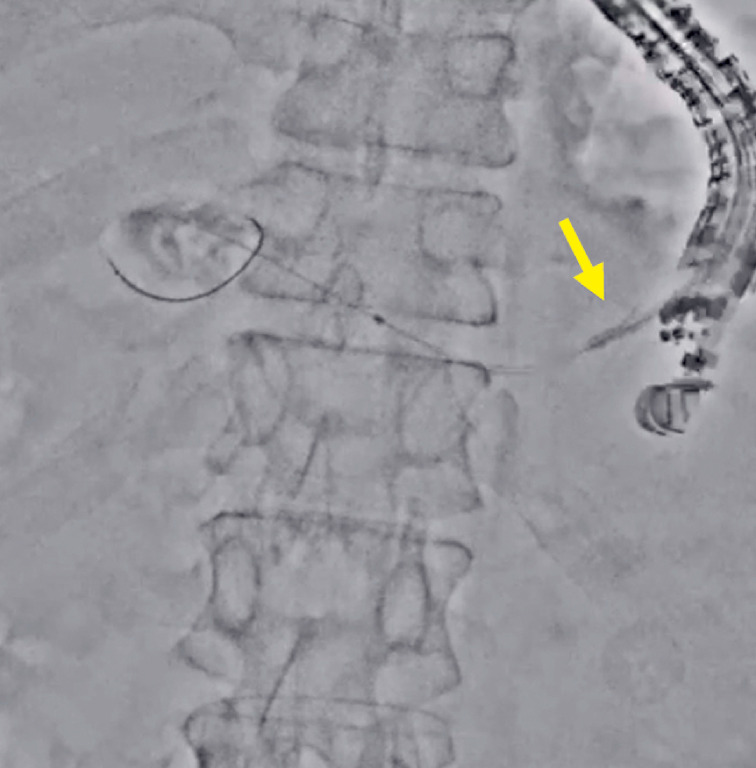
After switching Accent modes, the visibility of the plastic stent improved (arrow).

The guidewire can be observed in Accent mode, a novel visibility enhancement mode.Video 1

In conclusion, the newly available fluoroscopy mode can be used to enhance the visibility of devices that are often difficult to see during EUS-PDD.

Endoscopy_UCTN_Code_TTT_1AR_2AK

## Notes

Endoscopy E-Videos https://eref.thieme.de/e-videos
*E-Videos* is an open access online section of the journal *Endoscopy* , reporting on interesting cases and new techniques in gastroenterological endoscopy. All papers include a high-quality video and are published with a Creative Commons CC-BY license. Endoscopy E-Videos qualify for HINARI discounts and waivers and eligibility is automatically checked during the submission process. We grant 100% waivers to articles whose corresponding authors are based in Group A countries and 50% waivers to those who are based in Group B countries as classified by Research4Life (see: https://www.research4life.org/access/eligibility/ ). This section has its own submission website at https://mc.manuscriptcentral.com/e-videos .
